# Personalized Nutrition in Patients with Type 2 Diabetes and Chronic Kidney Disease: The Two-Edged Sword of Dietary Protein Intake

**DOI:** 10.3390/jpm12020300

**Published:** 2022-02-17

**Authors:** Milou M. Oosterwijk, Gerjan Navis, Stephan J. L. Bakker, Gozewijn D. Laverman

**Affiliations:** 1 Ziekenhuis Groep Twente, Department of Internal Medicine/Nephrology, 7609 PP Almelo, The Netherlands; g.laverman@zgt.nl; 2 Department of Internal Medicine, Division of Nephrology, University of Groningen, University Medical Center Groningen, 9713 GZ Groningen, The Netherlands; g.j.navis@umcg.nl (G.N.); s.j.l.bakker@umcg.nl (S.J.L.B.); 3 Department of Biomedical Signals and Systems, Faculty of Electrical Engineering, Mathematics and Computer Science, University of Twente, 7522 NB Enschede, The Netherlands

**Keywords:** type 2 diabetes, chronic kidney disease, dietary protein intake, sarcopenia

## Abstract

In type 2 diabetes (T2D), there is a general and strong focus on carbohydrate restriction. However, this may have unwarranted consequences for those with concomitant chronic kidney disease (CKD) since decreasing intake of carbohydrates implies a higher proportion of dietary protein, which is of critical debate in patients with CKD due to its ambiguous implications in maintaining either kidney function or nutritional status. We evaluated adherence to the protein recommendations, taking into account the nutritional status of patients with T2D with or without CKD. Patients were divided in three groups according to their estimated Glomerular Filtration Rate (eGFR): mild to no CKD (eGFR > 60 mL/min/1.73 m^2^), moderate CKD (eGFR 30–60 mL/min/1.73 m^2^), or advanced CKD (eGFR < 30 mL/min/1.73 m^2^). Regarding adherence to the protein recommendations, 17% of the patients without advanced CKD consumed < 0.8 g/kg/day, 29% of the patients with moderate CKD consumed > 1.3 g/kg/day, and 60% of the patients with advanced CKD consumed > 1.0 g/kg/day. In addition, patients with moderate- or advanced CKD tend to have a lower muscle mass, normalized by height, compared to patients with mild to no CKD (*p* < 0.001), while body mass index was not significantly different between patients with or without CKD (*p* = 0.44). We found that although dietary protein restriction has not been indicated in either of the CKD stages, approximately 10% had a dietary protein intake < 0.8 g/kg/day, with accompanying risks of malnourishment and sarcopenia. Our main advice is to maintain a dietary protein intake of at least 0.8 g/kg/day in order to prevent patients from becoming malnourished and sarcopenic.

## 1. Introduction

The population of patients with type 2 diabetes (T2D) is heterogeneous in various ways, and this may have implications for the nutritional needs [1]. About 30–40% of people with T2D, for example, develop chronic kidney disease (CKD) [2]. In T2D, there is a general and strong focus on carbohydrate restriction [3,4]. However, this may have unwarranted consequences for those with concomitant CKD since decreasing intake of carbohydrates implies a higher proportion of dietary protein, a nutrient which is of critical debate in patients with CKD due to its ambiguous implications in maintaining either kidney function or nutritional status [5,6].

The recommended daily allowance for adults with T2D as proposed by the American Diabetes Association equals an intake of 1.0–1.5 g/kg ideal body weight/day [7]. Regarding CKD, patients with moderate CKD (estimated Glomerular Filtration Rate (eGFR) < 60 mL/min/1.73 m^2^) are advised to avoid elevated dietary intakes of protein (>1.3 g/kg/day) [8]. Still, patients with advanced CKD (eGFR < 30 mL/min/1.73 m^2^) are advised to reduce dietary protein intake to 0.8 g/kg/day [8,9].

It has very recently been found in T2D that higher dietary protein intake is not associated with faster renal function deterioration, which applies to the full range of kidney function [10]. A beneficial association between dietary protein intake and development of CKD has also been previously found in the Ongoing Telmisartan Alone and in combination with Ramipril Global Endpoint Trial (ONTARGET) [11]. This renal safety of high protein intake is in contrast with the notion of stimulation of progression of kidney disease by high-protein diets [12]. This sheds new light on the management of dietary protein intake since the risk of malnutrition and sarcopenia as a result of low-protein diets in patients with CKD is of great concern [13]. 

In this paper, we investigate the adherence to the protein recommendations in a cohort study of patients with T2D with or without CKD and assess the nutritional status of these patients. Then, based on the findings, we discuss and personalize the dietary protein recommendations for patients with T2D with or without CKD.

## 2. Materials and Methods

### 2.1. Study Design

We performed a study in The Diabetes and Lifestyle Cohort Twente (DIALECT), a real-world observational cohort study in patients with T2D treated in secondary care in the Netherlands [14]. The cohort was set up to investigate the short- and long-term effects of lifestyle habits in people with T2D who received routine care and received no study-related interventions. The study has been approved by local institutional review boards (METC-Twente, NL57219.044.16; METC-Groningen, 1009.68020), is registered in The Netherlands Trial Register (NTR trial code 5855), and was performed according to the Guidelines of Good Clinical Practice and the Declaration of Helsinki.

### 2.2. Population

The study population consists of 433 patients with type 2 diabetes aged > 18 years. Patients depending on renal replacement therapy or patients with inability to understand the concept of informed consent were excluded from participation. For the current study, we excluded patients with missing objective dietary protein intake (*n* = 42), missing or incomplete subjective data on physical activity (*n* = 26), and missing subjective data on dietary intake (*n* = 4), leaving 361 patients for analysis.

### 2.3. Renal Function

Renal function is assessed by the estimated Glomerular Filtration Rate (eGFR) using the Chronic Kidney Disease Epidemiology Collaboration (CKD-EPI) formula [15]. We used cystatin-C based eGFR to define mild to no CKD (eGFR > 60 mL/min/1.73 m^2^), moderate CKD (eGFR 30–60 mL/min/1.73 m^2^) or advanced CKD (eGFR < 30 mL/min/1.73 m^2^) since creatinine-based eGFR may over- or underestimate kidney function in response to altering muscle mass [16].

### 2.4. Dietary Assessment

Objective total protein intake (g/day) was determined by the Maroni formula: 6.25 × ((0.0276 × urinary urea excretion (mmol/24-h)) + (0.031 × body weight)) + urinary protein excretion [17]. Ideal body weight was used to estimate total protein intake in g/kg/day, based on a BMI of 25 kg/m^2^, corresponding with current nutritional recommendations. 

Total energy intake was determined using a semi-quantitative Food Frequency Questionnaire (FFQ), which has been described extensively elsewhere [18].

### 2.5. Assessment of Nutritional Status

Body mass index (BMI) was calculated as weight divided by squared height (kg/m^2^). Muscle mass was estimated by 24-h urinary creatinine excretion rate (CER, mmol/24-h), which directly reflects functional metabolic muscle mass independent of kidney function [19]. Patients were asked to collect their 24-h urine to obtain the urinary CER by multiplying these concentrations with the volume of the 24-h urine collection. Patients were instructed to store the canister in a dark cool place, preferably in a refrigerator. To account for differences in muscle mass due to height differences, analyses were performed with CER normalized by height (CER/m^2^) [20].

Physical activity was subjectively assessed by the previously validated Short Questionnaire to Assess Health enhancing Physical Activity (SQUASH) [21]. We scored which patients meet the Dutch Healthy Exercise Norm of at least 30-min moderate-intensity activity a day for at least 5 days a week [22]. Other study procedures have been described extensively elsewhere [14,23]. 

### 2.6. Statistical Analysis

All cross-sectional statistical analyses were performed using SPSS version 27.0 (IBM, Chicago, Illinois). Normally distributed variables are presented as mean ± standard deviation and dichotomous variables as numbers (percentage). A two-tailed *p*-value < 0.05 was considered statistically significant.

Total protein intake was categorized into four groups: <0.8 g/kg/day, 0.8 to 1.0 g/kg/day, 1.0 to 1.3 g/kg/day, and >1.3 g/kg/day. Adherence to protein recommendations was defined as 1.0–1.5 g/kg/day (mild to no CKD), 0.8–1.3 g/kg/day (moderate CKD), and 0.8–1.0 g/kg/day (advanced CKD). Differences in characteristics between categories of CKD were tested by using the one-way ANOVA for normally distributed variables and the chi-square test for dichotomous variables.

## 3. Results

In the DIALECT population (*n* = 361), the mean age was 63 ± 9 years (Table 1). The population was obese, with a mean BMI of 32.7 ± 5.7 kg/m^2^, and only 5% had a BMI lower than 25 kg/m^2^. One-third of the patients (32%) suffered from renal function impairment (eGFR < 60 mL/min/1.73 m^2^), of which 4% suffered from advanced CKD (eGFR < 30 mL/min/1.73 m^2^). Mean dietary protein intake was 1.22 ± 0.33 g/kg/day (based on urea excretion data). A proportion of 10% of the patients had an intake below 0.8 g/kg/day, and more than one-third of the patients (38%) had an intake above 1.3 g/kg/day. 

### 3.1. Adherence to the Protein Recommendations

**Figure 1:**
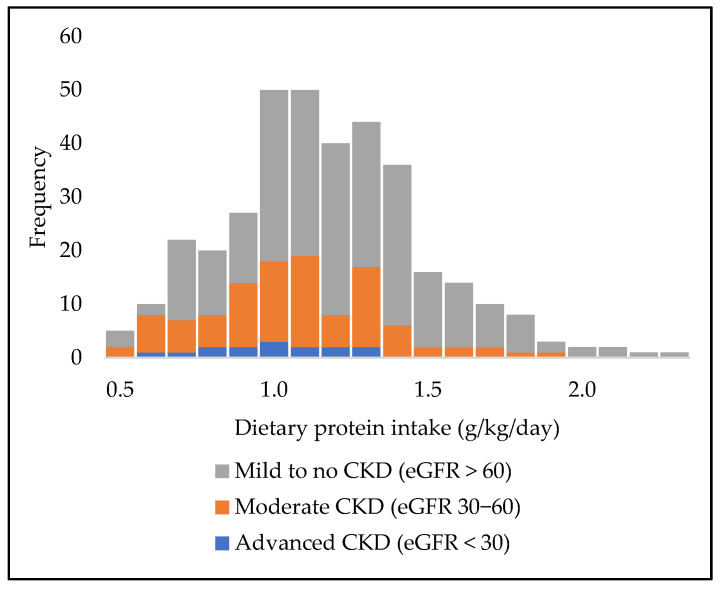
Distribution of dietary protein intake across stages of chronic kidney disease in the Diabetes and Lifestyle Cohort Twente.

Mean dietary protein intake was significantly higher in patients with mild to no CKD compared to patients with moderate or advanced CKD (*p* < 0.001). Dietary protein intake was not statistically different between patients with moderate or advanced CKD (*p* = 0.70) (Figure 1). Interestingly, the mean dietary intake in patients with advanced CKD was quite higher than the RDA of reducing dietary protein intake to 0.8 g/kg/day. 

In the patients with mild to no CKD, only 8% had a dietary protein intake < 0.8 g/kg/day, and this percentage was significantly lower compared to patients with moderate (15%) and advanced CKD (13%) (Figure S1a). In addition, in the patients with mild to no CKD, a considerable percentage achieved a dietary protein intake > 1.3 g/kg/day (43%), which was a significantly higher proportion than in the patients with moderate or advanced CKD. Although somewhat lower than in patients with mild to no CKD, still almost one-third (29%) of the patients with renal function impairment consumed > 1.3 g/kg/day despite the recommendation to avoid elevated dietary protein intake.

Regarding adherence to the guidelines, almost two-thirds of patients with T2D with or without CKD adhered to the protein recommendations (59%). However, 17% of the patients without advanced CKD still consumed < 0.8 g/kg/day, 29% of the patients with moderate CKD consumed > 1.3 g/kg/day, and even 60% of the patients with advanced CKD consumed > 1.0 g/kg/day (Figure S1b). 

Patients with moderate or advanced CKD not only had a lower protein intake, but they also had a lower energy intake (1851 ± 588 kcal/day) compared to patients with mild to no CKD (2075 ± 704 kcal/day). Moreover, patients who consumed less protein than recommended according to protein guidelines had a significantly lower energy intake compared to patients who consumed more protein than recommended according to protein guidelines (1841 ± 707 kcal/day versus 2118 ± 678 kcal/day, respectively). Furthermore, the mean dietary protein intake decreased across increasing stages of CKD even after adjustment for energy intake. Therefore, from the muscle perspective, increasing energy intake would be an item to consider. However, this obviously appears rather counterintuitive since 66% of patients with T2D included in our study suffer from obesity (BMI > 30 kg/m^2^).

### 3.2. Assessment of Nutritional Status

Regarding nutritional status in patients with T2D, a low muscle mass was not easily recognized because most were obese. The BMI was not different between patients with or without CKD (Figure S2). Regarding body composition, we saw a clear association with CKD. Patients with moderate or advanced CKD had a significantly lower muscle mass, estimated by 24-h urinary creatinine excretion and normalized by height, compared to patients with mild to no CKD.

Regarding physical activity, patients with mild to no CKD also had a higher trend towards adherence to the Dutch Healthy Exercise Norm of 30-min moderate-intensity activity a day for at least 5 days a week (62%) compared to patients with moderate or advanced CKD (50%) (Figure S3).

## 4. Discussion

In this study among 361 patients with T2D with or without CKD, we investigated adherence to the protein recommendations, and we assessed nutritional status of these patients. The main findings were that adherence to dietary protein intake is not optimal: non-adherence to the recommended guidelines for dietary protein also occurs in patients with mild to no CKD who did not receive any specific dietary advice to reduce dietary protein intake. In addition, patients with moderate or advanced CKD have significantly lower muscle mass compared to patients with mild to no CKD.

Personalization of dietary protein recommendations, taking CKD into account, seems to be of importance. For many years, dietary protein intake in patients with CKD has been an important topic of critical debate [24]. Dietary protein has traditionally been implicated as a factor fuelling progressive impairment of renal function in CKD [25]. High-protein diets may induce glomerular hyperfiltration and may lead to accumulation of toxic protein metabolites, while low-protein diets are adopted as part of a treatment aimed to promote kidney longevity [26]. Therefore, in the past, low-protein diets of 0.6–0.8 g/kg/day were advised in patients with CKD. Later literature in the early 2000s has indicated that long-term consumption of low-protein diets (<0.8 g/kg/day) did not conclusively result in delayed progression CKD [27], while it is strongly associated with elevated risk of sarcopenia [28].

Based on the current guidelines, there is no advice to restrict dietary protein in earlier stages of CKD. Therefore, in the earlier CKD stages, a liberal protein intake can be part of an approach that also includes elements such as muscle exercise in order to prevent the unwarranted process of muscle mass decline and physical inactivity. This approach fits very well in a diet with restriction of carbohydrates. The current guidelines do, however, advice to reduce dietary protein intake to 0.8 g/kg/day for persons with more advanced CKD stages (eGFR < 30 mL/min/1.73 m^2^). This advice is a trade-off since intake below this level carries the risk of sarcopenia, whereas higher levels have traditionally been considered as likely unfavourable for long-term kidney function. In clinical practice, to reach such a rather specific target for protein intake, it is strongly recommended to refer these patients with advanced CKD for counselling by a dietician, especially to prevent malnutrition. It is important to mention that recent findings from the observational DIALECT cohort demonstrated that a higher dietary protein intake, with an average of 1.22 ± 0.33 g/kg/day in persons with T2D, was not associated with faster renal function deterioration [10], and these results apply to the full range of kidney function. It appears, therefore, that when counselling these patients, it is more important to avoid protein intake from being too low, rather than too high, and that it is rather safe for long-term kidney function to accept higher protein intake.

Our main recommendation therefore would be to avoid a too-low dietary protein intake and to emphasize that a dietary protein intake of at least 0.8 g/kg/day should be maintained in order to avoid malnourishment in patients with T2D. Further research is needed to assess prospective associations between dietary protein intake and risk on sarcopenia in patients with advanced CKD. Still, it should be emphasized that the DIALECT data does not identify a safe upper limit of dietary protein intake, and therefore, caution for extremely high intakes of dietary protein is still in place. This is especially the case for patients with moderate CKD, of which 29% had a dietary protein intake above the recommendation. Since dietary counselling regarding CKD is limited to the pre-dialysis clinic (i.e., patients with eGFR < 30 mL/min/1.73 m^2^), it is unlikely that patients in the cohort have previously received dietary counselling aimed at protein restriction. This might reflect diet adaptations related to diabetes, emphasizing restriction of carbohydrates, which naturally implies a higher proportion of dietary protein. Therefore, this emphasizes the need for dietary counselling at earlier stages of CKD.

It should be realized, however, that dietary recommendations may have unwarranted effects. Particularly, when patients are warned not to eat more than a certain amount of protein per day, this could lead to decreased protein intake at levels lower than intended and thus increase the risk of malnutrition. This should lead to concern, especially regarding nutritional status of patients with CKD, since the risk of sarcopenia increases progressively with lower eGFR [13,29]. A dysfunctional metabolic state of patients with CKD may result in unwarranted accelerated muscle protein catabolism, which is in turn associated with adverse clinical outcomes [30]. On top of the elevated risk on premature mortality due to CKD, patients with CKD and concomitant sarcopenia may have an additional elevated risk on adverse clinical outcomes [31]. Therefore, in order to improve quality of life and longevity, it is of utmost importance to counteract sarcopenia in patients with CKD.

Our results indicate that the combination of T2D with CKD is accompanied by a clustering of lower dietary protein intake, less physical activity, and low muscle mass, while BMI is equally high in the obesity range. As expected, patients with mild to no CKD have significantly higher muscle mass compared to patients with moderate or advanced CKD. This is consistent with a higher prevalence of sarcopenic obesity in patients with moderate or advanced CKD, which was previously assessed in the general T2D population [23,32]. From the muscle perspective, increasing energy intake would be an item to consider next to increasing dietary protein intake. However, this obviously appears rather counterintuitive since two-thirds of patients with T2D included in our study suffer from obesity.

With respect to body weight, we have previously demonstrated that the patients in the DIALECT cohort had a BMI that was very stable over a timespan of several decades, somewhat in contrast with the notion that body weight in middle-aged people gradually increases over the years [33]. An explanation might be found in the extensive literature showing that a gradual loss of muscle mass occurs in middle-aged people with increasing age, even accelerating at older ages [34,35]. Therefore, we assume that in our population, changes in body composition do occur in which a loss of muscle mass may be masked by an increase in fat mass, whereby fat mass was proven to be continued to increase until 75 years of age [36]. Therefore, it would be worthwhile to assess changes in body composition rather than BMI in order to early detect and effectively counteract sarcopenia.

As stated, counselling of patients with CKD by a dietician is indicated in order to assess overall nutritional status [37], which becomes relevant already at a stage of moderate severity. As part of this, it is valuable to assess their actual current dietary protein intake. This enables to reduce dietary protein only in persons with excessive levels of dietary protein intake and prevent iatrogenic malnutrition. Next to assessment of dietary protein intake in combination with other macronutrients and total energy intake, secondary causes of malnutrition (e.g., decreased appetite, bad nutritional habits, or incorrect application of generalized nutritional advices received by health care professionals) are taken into account.

It is well known that for maintaining or enhancing muscle mass, patients should not only increase dietary protein intake but also enhance physical activity [38]. Therefore, a personalized approach for dietary protein intake also includes an evaluation of physical fitness, i.e., muscle mass and physical activity level. We have previously found a clear association between dietary protein intake and muscle mass in the overall T2D population in the DIALECT cohort and also a clear association between muscle mass and physical activity [23,32]. This suggests that assessment of muscle mass and physical activity may be useful in assessment of the prevalence and severity of sarcopenia. Since patients with CKD are more likely to suffer from sarcopenia, this emphasizes the importance of enhancing physical activity in patients with CKD in order to maintain muscle mass. In the general T2D population, there are a great many opportunities to enhance physical activity up to at least 30-min of moderate-intensity activity a day since only 58% of the DIALECT patients adhere to the guideline for physical activity [22]. Therefore, recent recommendations also focus on physical activity or exercise, preferably undertaken daily by all older people, for as long as possible [39,40].

Preferably, dietary protein intake, muscle mass, and physical activity level should be regularly assessed and monitored in routine clinical care, and we did this by collection of 24-h urine samples. An alternative, possibly less burdensome method could be assessment of physical activity and muscle strength by assessment of hand-grip strength or walk speed. Finally, it would be worthwhile to define age-specific ranges of low, intermediate, and high values for muscle mass in order to recognize sarcopenia. We have previously found in the DIALECT cohort that the 24-h urinary creatinine excretion rate could be used as an indicator of either muscle mass in routine clinical care and that it even is an indicator of physical activity [41].

In summary, in the treatment of persons with T2D, it is important not to overlook dietary protein intake. For dietary counselling, an individual assessment is required, taking into account patient characteristics, such as current protein intake, presence of CKD, muscle mass, and physical activity. Patients with CKD, even at moderate stage, are at increased risk of sarcopenia and low physical activity. In these patients, the emphasis should be on not going too low with protein intake, especially since recent evidence indicates that higher protein intake is not accompanied by renal function deterioration, as was previously assumed. 

## Figures and Tables

**Table 1:** Baseline patient characteristics by categories of chronic kidney disease of 361 patients with type 2 diabetes in the Diabetes and Lifestyle Cohort Twente.

	Total Population	Mild to No CKD (eGFR > 60)	Moderate CKD (eGFR 30−60)	Advanced CKD (eGFR < 30)	*p*-Value
*n*	361	246 (68)	100 (28)	15 (4)	
Age (years)	63 ± 9	61 ± 9	68 ± 7	70 ± 7	<0.001
Sex (male)	209 (58)	149 (61)	50 (50)	10 (67)	0.15
BMI (kg/m^2^)	32.7 ± 5.7	32.6 ± 5.7	33.2 ± 5.7	30.6 ± 4.8	0.22
Normal weight (BMI < 25)	18 (5)	15 (6)	2 (2)	1 (7)	0.44
Overweight (BMI 25–30)	105 (29)	68 (28)	31 (31)	6 (40)	
Obese (BMI > 30)	238 (66)	163 (66)	67 (67)	8 (53)	
Muscle mass (CER/m^2^)	4.64 ± 1.40	4.84 ± 1.42	4.28 ± 1.28	3.93 ± 1.00	<0.001
Energy intake (kcal/day)	2004 ± 677	2075 ± 704	1818 ± 584	2072 ± 589	0.005
Dietary protein intake (g/kg/day)	1.22 ± 0.33	1.27 ± 0.33	1.12 ± 0.29	1.05 ± 0.23	<0.001
<0.8 g/kg/day	37 (10)	20 (8)	15 (15)	2 (13)	0.017
0.8–1.0 g/kg/day	47 (13)	25 (10)	18 (18)	4 (27)	
1.0–1.3 g/kg/day	140 (39)	95 (39)	38 (38)	7 (47)	
>1.3 g/kg/day	137 (38)	106 (43)	29 (29)	2 (13)	
Adherence to protein recommendations					
Above recommendations	62 (17)	45 (18)	15 (15)	2 (13)	0.006
According to recommendations	212 (59)	152 (62)	56 (56)	4 (27)	
Below recommendations	87 (24)	49 (20)	29 (29)	9 (60)	
Adherence to the Dutch Healthy Exercise Norm					
No adherence	150 (42)	93 (38)	51(51)	6 (40)	0.08
Adherence	211 (58)	153 (62)	49 (49)	9 (60)	

Continuous variables are presented as mean ± standard deviation and dichotomous variables as numbers (percentage). BMI, body mass index; CER, 24-h urinary creatinine excretion; CKD, chronic kidney disease; eGFR, estimated Glomerular Filtration Rate.

## Data Availability

The authors will consider every reasonable request to inspect the data used for this article.

## Supplementary Materials

The following are available online at https://www.mdpi.com/article/10.3390/jpm12020300/s1, Figure S1: (**a**) Categories of dietary protein intake (g/kg/day) across stages of chronic kidney disease (CKD) in the Diabetes and Lifestyle Cohort Twente; (**b**) Adherence to protein recommendations across stages of CKD in the Diabetes and Lifestyle Cohort Twente, Figure S2: Categories of body mass index (BMI, kg/m2) across stages of chronic kidney disease (CKD) in the Diabetes and Lifestyle Cohort Twente, Figure S3: Adherence to the Dutch Healthy Exercise Norm across stages of chronic kidney disease (CKD) in the Diabetes and Lifestyle Cohort Twente.
