# Supplementary material for: Personalized Nutrition in Patients with Type 2 Diabetes and Chronic Kidney Disease: The Two-Edged Sword of Dietary Protein Intake
Source: J Pers Med. 2022 Feb 17;12(2):300. doi: 10.3390/jpm12020300 (PMC8878174; doi:10.3390/jpm12020300)
Supplement: Supplementary file 1 [file jpm-12-00300-s001.zip › jpm-1515589-supplementary.pdf]

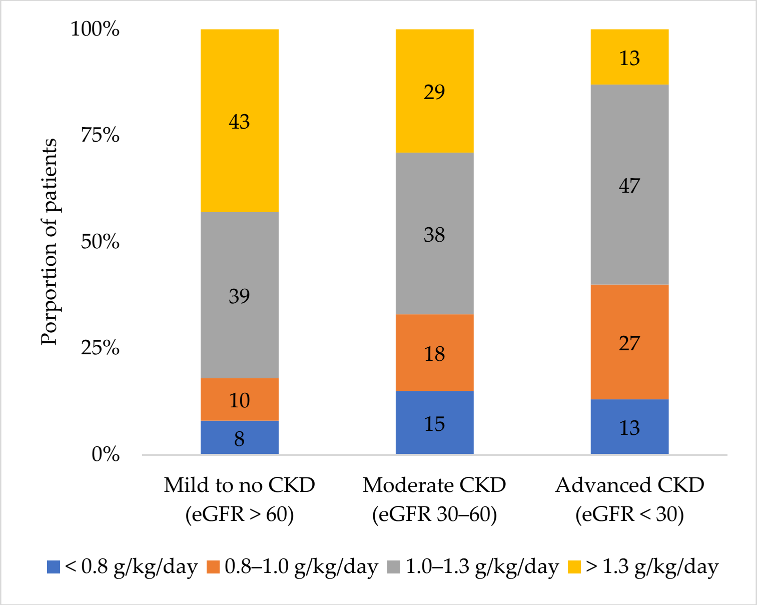

(**a**)

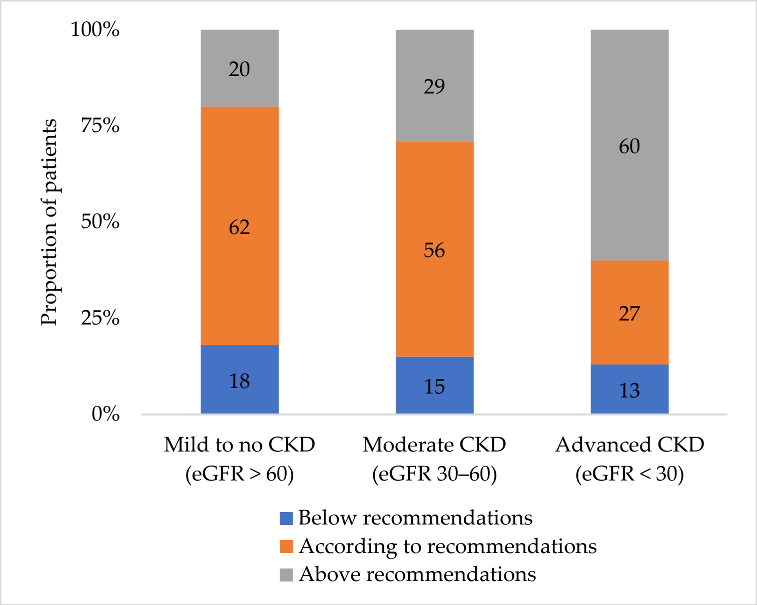

(**b**)

Figure S1. (**a**) Categories of dietary protein intake (g/kg/day) across stages of chronic kidney disease (CKD) in the Diabetes and Lifestyle Cohort Twente; **(b)** Adherence to protein recommendations across stages of CKD in the Diabetes and Lifestyle Cohort Twente.

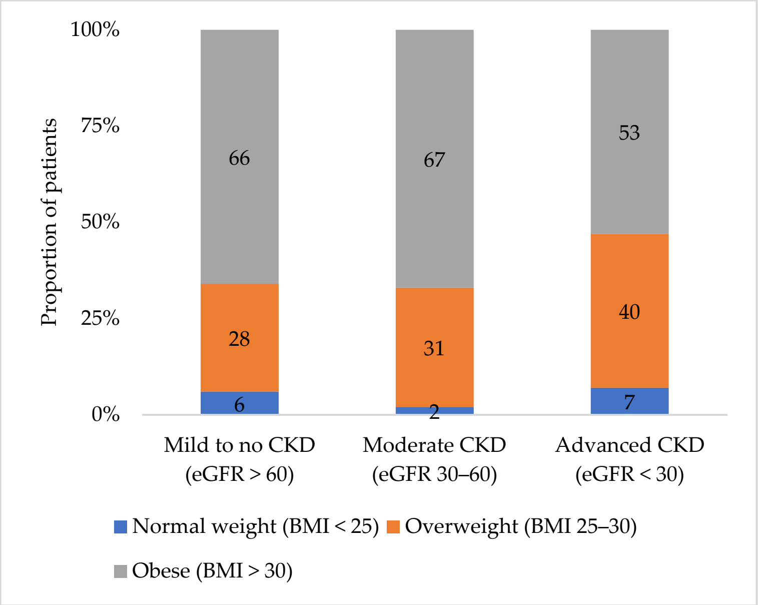

Figure S2. Categories of body mass index (BMI, kg/m^2) across stages of chronic kidney disease (CKD) in the Diabetes and Lifestyle Cohort Twente.

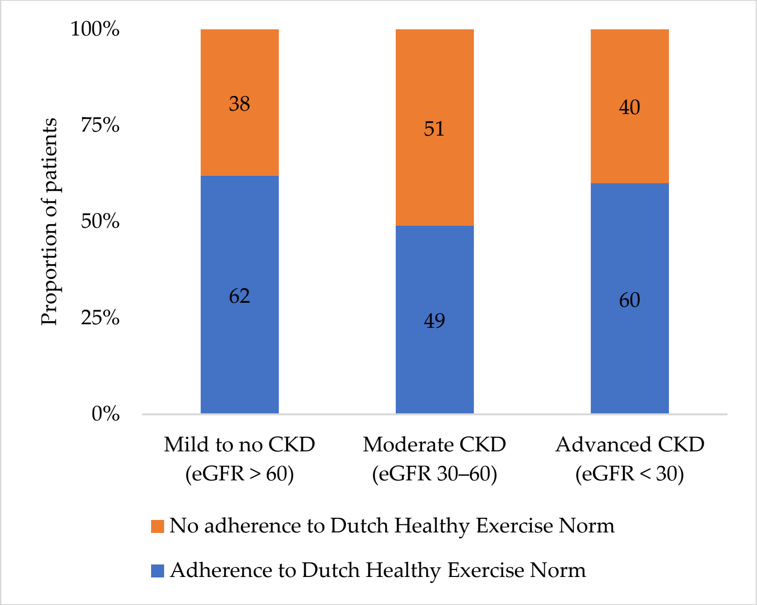

Figure S3. Adherence to the Dutch Healthy Exercise Norm across stages of chronic kidney disease (CKD) in the Diabetes and Lifestyle Cohort Twente.
